# The Impacts of the Sterilization Method and the Electrospinning Conditions of Nanofibrous Biodegradable Layers on Their Degradation and Hemocompatibility Behavior

**DOI:** 10.3390/polym16081029

**Published:** 2024-04-09

**Authors:** Kristyna Havlickova, Eva Kuzelova Kostakova, Maxim Lisnenko, Sarka Hauzerova, Martin Stuchlik, Stanislava Vrchovecka, Lucie Vistejnova, Jiri Molacek, David Lukas, Renata Prochazkova, Jana Horakova, Sarka Jakubkova, Bohdana Heczkova, Vera Jencova

**Affiliations:** 1 Department of Chemistry, Faculty of Science, Humanities and Education, Technical University of Liberec, Studentská 1402/2, 46117 Liberec, Czech Republic; maxim.lisnenko@tul.cz (M.L.); sarka.hauzerova@tul.cz (S.H.); david.lukas@tul.cz (D.L.); vera.jencova@tul.cz (V.J.); 2 Institute for Nanomaterials, Advanced Technology and Innovation, Technical University of Liberec, Bendlova 1409/7, 46117 Liberec, Czech Republic; martin.stuchlik@tul.cz (M.S.); stanislava.vrchovecka@tul.cz (S.V.); 3 Biomedical Center, Faculty of Medicine in Pilsen, Charles University, Alej Svobody 1655/76, 32300 Pilsen, Czech Republic; lucie.vistejnova@lfp.cuni.cz (L.V.); molacek@fnplzen.cz (J.M.); 4 Department of Surgery, Faculty of Medicine in Pilsen, Charles University, Alej Svobody 80, 32300 Pilsen, Czech Republic; 5 Regional Hospital Liberec, Husova 357/28, 46001 Liberec, Czech Republic; renata.prochazkova@nemlib.cz (R.P.); sarka.marikova@nemlib.cz (S.J.); bohdana.heczkova@nemlib.cz (B.H.); 6 Institute of Clinical Disciplines and Biomedicine, Faculty of Health Studies, Technical University of Liberec, Studentská 1402/2, 46117 Liberec, Czech Republic; 7 Department of Nonwovens and Nanofibrous Materials, Faculty of Textile Engineering, Technical University of Liberec, Studentská 1402/2, 46117 Liberec, Czech Republic; jana.horakova@tul.cz

**Keywords:** electrospun nanofibers, biodegradable polyester, sterilization, gamma irradiation, ethylene oxide, enzymatically catalyzed degradation, hemocompatibility

## Abstract

The use of electrospun polymeric biodegradable materials for medical applications is becoming increasingly widespread. One of the most important parameters regarding the functionality of nanofiber scaffolds during implantation and the subsequent regeneration of damaged tissues concerns their stability and degradation behavior, both of which are influenced by a wide range of factors (the properties of the polymer and the polymer solution, the technological processing approach, the sterilization method, etc.). This study monitored the degradation of nanofibrous materials fabricated from degradable polyesters as a result of the sterilization method applied (ethylene oxide and gamma irradiation) and the solvent system used to prepare the spun polymer solution. Aliphatic polyesters PCL and PLCL were chosen for this study and selected with respect to the applicability and handling in the surgical setting of these nanofibrous materials for vascular bandaging. The results revealed that the choice of solvent system exerts a significant impact on degradation during sterilization, especially at higher gamma irradiation values. The subsequent enzyme-catalyzed degradation of the materials following sterilization indicated that the choice of the sterilization method influenced the degradation behavior of the materials. Whereas wave-like degradation was evident concerning ethylene oxide sterilization, no such behavior was observed following gamma-irradiation sterilization. With concern for some of the tested materials, the results also indicated the potential for influencing the development of degradation within the bulk versus degradation from the surface of the material. Both the sterilization method and the choice of the spinning solvent system were found to impact degradation, which was observed to be most accelerated in the case of PLCL (L-lactide-co-caprolactone copolymer) electrospun from organic acids and subsequently sterilized using gamma irradiation. Since we planned to use these materials in cardiovascular applications, it was decided that their hemocompatibility would also be tested. The results of these tests revealed that changes in the structures of the materials initiated by sterilization may exert thrombogenic and anticoagulant impacts. Moreover, the microscopic analysis suggested that the solvent system used in the preparation of the materials potentially affects the behavior of erythrocytes; however, no indication of the occurrence of hemolysis was detected.

## 1. Introduction

Various nanofibrous materials, which have significant potential for application in the field of tissue engineering in particular [1], are currently being developed for use in medical applications. The structure of these materials mimics that of the natural extracellular matrix (ECM), of which polymeric nanofibers form an essential part. Due to their specific morphology, nanofibrous materials have properties (especially high porosity and extensive active surfaces—“surface to volume ratio”) that allow for the adsorption of proteins and subsequent cell adhesion and proliferation [2]. This structure is also important in terms of the transport of nutrients and metabolic waste. With respect to the target tissue, such materials can be prepared from various polymers with specific structures, and, concerning their final application (the target tissue), the appropriate choice of polymer and material preparation technique is of paramount importance. The use of biodegradable materials appears to be promising for cardiovascular applications, including vascular prostheses and vascular bandages [3,4,5,6,7,8]. Since such materials perform their functions on a temporary basis, it is not necessary to remove them following the repair of the tissue. However, it is important to ensure a suitable degradation rate that guarantees that the material performs its function reliably (especially concerning its mechanical properties) over the necessary period of time [9]. Furthermore, the use of biodegradable polymers is advantageous from the point of view of the potential incorporation of biologically active substances, which can be released in a controlled manner via both the diffusion process and the degradation of the polymer [3,8,10]. Both natural (e.g., collagen and gelatin) and synthetic (most often aliphatic polyesters) biodegradable polymers can be applied. Synthetic (rather than natural) polymers are often used in medical applications in which it is necessary to ensure an identical material structure and, thus, the stability of the resulting materials. The most commonly used synthetic materials comprise FDA-approved aliphatic polyesters, e.g., polycaprolactone (PCL), polylactic acid (PLA), and polyglycolic acid (PGA), and their copolymers PLGA (a copolymer of lactic acid and glycolic acid) and PLCL (a copolymer of lactic acid and caprolactone) [11,12].

The two slowly degrading polyesters, PCL and PLCL, were selected for study as suitable materials for the preparation of biodegradable vascular bandages. Biocompatible hydrophobic PCL is used for the preparation of degradable nanofibrous materials for various applications, including vascular prostheses and bandages [4,5,8,13]. It evinces a long degradation time, thus ensuring good mechanical properties during the repair of tissue [14]. The PLCL copolymer has excellent mechanical properties, is less hydrophobic than PCL, has a slow degradation time, and is particularly suitable for cardiovascular applications [5,6,15]. In addition to the choice of polymer, the morphology of such materials may also be influenced by the technology used in the production process, as well as its technological parameters. The spinning process allows for the production of microfiber and/or nanofiber layers with a structure that is suitable for the target tissue. Electrospinning, i.e., one of the most intensively applied nanofiber-production processes, leads to the formation of nanofibers from a polymer liquid (solution or melt) as a result of electrical forces based on electrohydrodynamics [16]. The success of the process and the resulting parameters of the nanofibrous material depend on a number of factors. The process parameters (the type and construction of the spinning electrode; the type and construction of the collector; the distance between the spinning electrode and the collector; the type of electrical voltage source, i.e., direct current—DC or alternating current—AC and its frequency and signal shape [17]; the electric voltage and current applied; the dosing of the spinning liquid on the spinning electrode, etc.) and the material parameters (for polymer solutions—the types of polymer and solvents used; the viscosity (the concentration of the polymer in the solution); the molecular weight of the polymer; the surface tension; the electrical conductivity; the pH; additives, etc.) are responsible for the development and results of the electrospinning process [16,18]. The material conditions, i.e., the composition of the spun liquid and the polymeric solution, also play a crucial role in the determination of the optimal morphology and the internal arrangement of biodegradable polymer electrospun nanofibers. The degradation of polymer chains due to the solvents used, which leads to a decrease in the molecular weight, is, in most cases, undesirable in that it may lead to changes in the spinning process (such as a reduction in the viscosity of the solution and, thus, a reduction in the fiber diameters, reduced productivity, and even electrospraying). Therefore, the storage time and temperature of polymer solutions destined for electrospinning must be optimized according to the materials used. For example, carboxylic acids are considered to be “benign” or mildly toxic, thus making them ideal solvents for the PCL and PLCL biodegradable polyesters [19]. However, at laboratory temperature, the hydrolytic degradation of the aliphatic polyesters within PCL and PLCL occurs over time [20,21], and only a decrease in the acid concentration or a decrease in the temperature are able to suppress the progress of the degradation process.

In addition to the material and process conditions of the spinning procedure, the sterilization method and dose may also affect the internal structure of the nanofibers. Sterilization represents a key stage in the development of biomaterials for medical use since infection could lead to serious complications following implantation. The elimination of microorganisms (bacteria, yeasts, fungi, protozoa, spores, and viruses) is essential in terms of ensuring an acceptable level of safety for all materials that are implanted in the body. Although a wide range of sterilization methods are available, there is no “one solution that fits all” in terms of effectiveness and passivity concerning the properties of the material to be sterilized (Chausse et al., 2023; Dai et al., 2016; Dong et al., 2009; Krug et al., 2023) [22]. Materials made from biodegradable polymers are particularly sensitive in this regard; the sterilization process may lead to changes in the physicochemical properties as well as the biological identity of the scaffold [14,23,24,25]. Taking into account the chemical compositions of the materials employed in this study and based on previous results presented in the literature [24,26,27,28], we selected the low-temperature ethylene oxide (EO) and gamma irradiation (GAMMA) sterilization methods. EO (chemical) sterilization is particularly effective at low temperatures; however, this method entails the risk of chemical modification of the polymer, which could lead to changes in the mechanical properties, degradation behavior, and biocompatibility [26,27,29]. Although ionizing gamma irradiation sterilization is a very effective sterilization method (including for SARS-CoV-2 [30]), changes in the covalent structure of the polymer (the crosslinking of macromolecules and the cleavage of bonds) may lead to changes in the resulting properties of the polymer [24,31,32,33].

This study describes the impacts of these sterilization methods on nanofibrous materials made from biodegradable polyesters, particularly with regard to changes in their morphology, degradation behavior, and hemocompatibility. Although the basic impacts of various sterilization methods on electrospun PCL nanofibers with a molecular weight of 45 kDa have already been reported [27], the impacts of two differing doses of gamma irradiation have not yet been tested. Moreover, in contrast to previous comparable studies, our study considered a set of polymers including PCL with a molecular weight of 80 kDa and PLCL copolymer materials rather than just PCL of 45 kDa. The study also included the selection of suitable solvent systems with regard to the degradation of PLCL; whereas the spinning of PLCL from an organic acid solution results in changes in the morphology of the material, this approach has the potential to influence the degradation behavior of the resulting materials [34].

## 2. Materials and Methods

### 2.1. Materials

A copolymer of L-lactide and ε-caprolactone (PLCL) with a 70:30 molar ratio and molecular weight of Mn 80 kDa (determined by GPC analysis at the home institution, described in the Section 2.7. Purasorb PLCL 7015, was purchased from Corbion (Amsterdam, The Netherlands), and poly-ε-caprolactone with an average molecular weight of M_n_ 80 kDa and polycaprolactone with an average molecular weight of M_n_ 45 kDa (both purchased from Merck KGaA (Darmstadt, Germany)) were used as the polymer materials. The optimal type of PLCL was chosen according to the results of previous research [34]. The polymer materials were dissolved in the following solution systems: (i) acetic acid/formic acid/acetone at a weight ratio of 1:1:1; (ii) chloroform/ethanol at a weight ratio of 8:2. All the solvents were purchased from Penta (Prague, Czech Republic). The choice of the solution system that combined acetic acid, formic acid, and acetone was inspired by [35]. The final polymer concentrations were 10 wt% and 16 wt%. Table 1 provides a list of the four prepared solutions. The solvents tetrahydrofuran (THF) (for the PCL and PLCL) and chloroform (CHCl_3_) (for the PLLA) (Penta, Prague, Czech Republic) were used for the gel permeation chromatography analysis. Lipase in the form of *Pseudomonas cepacia* (30 ≥ U/mg; Merck KGaA (Darmstadt, Germany)) and Proteinase K (30 ≥ U/mg; Merck KGaA (Darmstadt Germany)) were used for enzymatic degradation experimentation purposes. The phosphate buffer (PBS; pH 7.4) consisted of 136 mmol/L sodium chloride (NaCl), 0.268 mmol/L potassium chloride (KCl), 0.01 mmol/L dibasic dodecahydrate sodium phosphate (Na_2_HPO_4_∙12H_2_O), 1.38 mmol/L monopotassium phosphate (KH_2_PO_4_) (Penta, Prague, Czech Republic), distilled water, and 3.08 mmol/L sodium azide (NaN_3_) as the antibacterial agent.

### 2.2. Electrospinning

All the materials were electrospun from freshly prepared solutions after 4 h using a magnetic stirrer. All the nanofibrous materials were DC electrospun on an NS 1WS500U needle-less electrospinning machine (Elmarco, Liberec, Czech Republic) based on a stationary wire spinning electrode. The electrospinning conditions had already been optimized during the preliminary experimentation phase, which allowed for the production of a macroscopically homogeneous nanofiber layer without any significant defects and with a sufficient degree of productivity. The spinning electrode was positively charged using a direct current high voltage source (40 kV), and the collector was negatively charged (−10 kV). The distance between the spinning electrode and the collector was set at 180 mm. The polymer solution was fed onto the stationary wire spinning electrode using a moving carriage module filled with the polymer solution. A metal insert orifice of 0.7 mm in diameter was used for the regulation of the layer of polymer solution on the spinning electrode. The DC electrospinning device was complemented by an air conditioning unit, which ensured the stability of the pre-determined temperature and air humidity values in the spinning space. The temperature was set at 22 ± 2 °C and the relative humidity at 40 ± 2%. The supporting material comprised 20 g/m^2^ spunbond Pegatex S (PFNonwovens, Znojmo, Czech Republic), and the withdrawal speeds were changed according to the values shown in Table 1 so as to obtain similar final areal weights for the various nanofibrous layers.

### 2.3. Sterilization

The experiment monitored the in vitro degradation of the biodegradable polyesters in the nanofibrous layers as a result of the application of the selected sterilization methods: ethylene oxide at room temperature (Anprolene model AN74, Andersen Sterilizer, Essex, United Kindom), gamma irradiation via a precision dose of 15 kGy ± 10% (result 14.7 kGy), and gamma irradiation via a precision dose of 40 kGy ± 10% (39.6 kGy) (Bioster, Veverská Bítýška, Czech Republis) as the maximum dose for samples with the potential for in vivo and clinical testing. The ethylene oxide sterilization process was performed according to the ISO 1135-1 standard [36]. The samples were sterilized at RT for 12 h in an Anprolene sterilizing device. The materials were then vented at room temperature for more than two weeks. The gamma irradiation sterilization was performed in compliance with ISO 11137-1 [37], ISO 11137-2 [38], ISO 11137-3 [39] standards. All the materials and samples were stored at room temperature and in darkness prior to the analysis.

### 2.4. Enzymatic Degradation

An accelerated degradation method was applied to evaluate the impact of sterilization on the degradation behavior of the materials.

Small samples cut from the sterilized electrospun layers (*n* = 3 for each material/1 testing day + 2 negative controls (NC)) were used for the accelerated enzymatic degradation experiments; the samples (with weights of 50 ± 1 mg) were emplaced in 5 mL vials. Enzymatic degradation was performed at 37 °C in PBS aimed at simulating biological fluids by using model enzymes to catalyze the cleavage of the ester bond in the polyesters. The resulting solutions contained 5 mL of PBS with lipase (30 ≥ U/mg; Merck KGaA, Darmstadt, Germany) at a concentration of 5 U/mL or proteinase K (30 ≥ U/mg; Merck KGaA, Darmstadt, Germany) at a concentration of 3 U/mL of enzymes; pure PBS only was used for the NC. It was not possible to use the same enzyme to catalyze the degradation of both the PCL and PLCL polymers (apparently due to substrate specificity; the available enzymes were not able to catalyze the reaction for both polymers). Hence, it was decided to use lipase from the bacterial strain *Pseudomonas cepacia* to degrade the PCL [40,41,42,43] whereas proteinase K from the fungus *Tritirachium album* was used to evaluate the degradation behavior of the PLCL copolymer [44]. The concentrations of the enzymes were set so that the degradation of the tested materials took approximately the same time (1 week). The PBS solution with enzymes was changed on a daily basis; three sample replicates were removed at the same time, washed twice with distilled water, and dried in a dryer at 25 °C for 2 days prior to further investigation. Following thorough drying, the samples were immediately weighed using digital scales (PA224C four-range analytical scales, Ohaus, Nänikon, Switzerland). The weights of the samples before and after degradation were recorded in order to determine the weight loss; the values were then entered into Equation (1):

(1) $$w_{loss}[\%]=\frac{w_{after}-w_{before}}{w_{before}}*100,$$

where *W_before_* is the dry weight (g) before degradation and *W_after_* is the dry weight (g) after degradation. Three replicate samples were prepared, and the resulting values were averaged.

### 2.5. Scanning Electron Microscopy

Electrospun samples with dimensions of 0.5 × 0.5 cm were sputtered with a thin layer of gold and imaged using a Vega 3SB Easy Probe (TESCAN, Brno, Czech Republic) scanning electron microscope. The fiber diameters (approximately 100 measurements for each sample) were measured using FIJI/ImageJ software (version number 2.1.0/1.53t, NIH).

### 2.6. Infrared Spectroscopy

The chemical compositions of the materials were investigated using a Fourier transform spectrometer (FTIR) (Nicolet iZ10; Thermo Fisher Scientific, Waltham, MA USA) at room temperature. The samples were placed on an ATR diamond crystal for analysis purposes, and the spectrum analysis was performed in the infrared region within the 4000–400 cm^–1^ range with a spectral resolution of 4 cm^–1^. Atmospheric and baseline corrections were subsequently applied.

### 2.7. Gel Permeation Chromatography

Gel permeation chromatography (GPC) was employed so as to observe changes in the molecular weight of the analyzed materials. Samples of 4 mg were dissolved in THF or chloroform and mixed using an automatic vortex mixer. The solutions were then filtrated using a 13 mm PTFE syringe microfilter (diameter of 13 mm, pore size of 0.45 μm). The analysis was performed using a Dionex UltiMate 3000 HPLC device (Thermo Fischer Scientific, Waltham, MA, USA), which included an Ultimate 3000 pump, an autosampler, and a column compartment. A Varian 385-LC Evaporative Light Scattering Detector (ELSD) was used for the detection of the polymers. A Phenogel column (300 mm; i.d. 7.8 mm; 5 μm; 10E5Å) was used for separation purposes. The mobile phase comprised tetrahydrofuran. The separation conditions were set as follows: a flow rate of 1 mL·min^−1^, the temperature of the column compartment at 35 °C, and a sample volume of 30 μL. The ELSD conditions for the detection of the polymers comprised a laser intensity of 10%, an evaporator temperature of 80 °C, a nebulizer temperature of 90 °C, and a drying gas-carried flow of 1 SML. The analysis lasted for 15 min.

### 2.8. Hemocompatibility

The interaction of the sterilized materials with blood components was investigated following the methodology described in a previous study [45] and is based on the standard [46]. Hemolysis was tested on 1 × 1 cm samples with a solution of fresh whole blood (4 mL of anticoagulated blood mixed with 5 mL of PBS buffer, pH 7.4). The 200 µL solution was added to 10 mL of PBS with the sample of the material (which was incubated in PBS for 30 min at 37 °C prior to the addition of the blood). Following incubation for 1 h at 37 °C with the blood solution, the test tubes were centrifuged (100× *g*, 20 min), and 100 μL of the supernatant was analyzed using spectrophotometry for the presence of free hemoglobin (at a wavelength of 570 nm). Distilled water (positive control, occurrence of hemolysis) and PBS (negative control, no occurrence of hemolysis) were used as the controls. The percentage of hemolysis was then calculated from the measured absorbance values by applying the following formula:

Hemolysis [%] = [(A_sample_ − A_neg_)/(A_pos_ − A_neg_) ∗ 100. (2)

The influence of the materials on platelet activation (the thrombogenicity test) was assessed as the viability of the platelets after 2 h of incubation with the material; the comparison was performed with the platelets only in the well of the microtiter plate (the positive control). 0.5 mL of the platelet solution (prepared at the Regional Hospital, Liberec; concentration of 600–900 × 10^9^ platelets/L, 1 day following donation) was added to the wells of a 48-well plate with the 1 × 1 cm samples (*n* = 10). After 2 h of incubation at 37 °C in a 5% CO_2_ atmosphere, the solution was aspirated, and the viability of the activated (adhered) platelets was measured using the following CCK test: 0.5 mL of 10% CCK-8 solution in intersol was added after 2 h of incubation (37 °C, 5% CO_2_); the absorbance of the products was measured at 450 nm. The influence of the materials on coagulation was evaluated employing two tests, i.e., the prothrombin time test (PT test) and the activated partial thromboplastin time test (aPTT test). Thawed clinical plasma (PPP—platelet pure plasma, Regional Hospital, Liberec, Czech Republic) was used for testing purposes. 500 μL of plasma was incubated with the 1 × 1 cm samples (*n* = 5) for 45 min at 37 °C. The PT and aPTT times were then measured using an analyzer (BCS XP, Siemens Healthineers, Forchheim, Germany) in accordance with the instructions provided with the device. Hemocompatibility was tested at different time intervals, i.e., up to 3 months and 1 year after sterilization.

### 2.9. Statistical Analysis

The quantitative data were presented as the mean with the standard mean error. The normality of the numerical data was verified using Shapiro–Wilk’s normality test. The statistical comparisons for the normally distributed data obtained from the hemocompatibility tests were performed using the one-way ANOVA analysis with the Tukey multiple comparisons test. The statistical comparisons of the non-normally distributed data were performed using nonparametric analysis and, subsequently, the Kruskal–Wallis test for multiple comparisons [45]. The statistical evaluation of the weight loss of the materials was assessed employing the Welch and Brown–Forsythe (ANOVA) test. The analysis was performed using GraphPad Prism 5 software (GraphPad Software, San Diego, CA, USA). A *p* value of *p* ≤ 0.05 was considered statistically significant.

## 3. Results and Discussion

This study analyzed the impacts of gamma irradiation and ethylene oxide sterilization on the degradation behavior and hemocompatibility of various polyester nanofibrous materials. The polyester materials comprised poly(ε-caprolactone) (PCL) and the L-lactide-co-caprolactone copolymer (PLCL). The materials were electrospun from differing solvent systems. The electrospun nanofibrous materials were sterilized using gamma irradiation and ethylene oxide methods. An analysis was subsequently performed of the impacts of the sterilization methods on selected properties of the materials—polymer degradation, changes in the chemical structure of the polymer, and changes in the morphology of the materials. In addition, this study included an investigation of the impacts of sterilization on the biological behavior of the materials, which included monitoring the changes in the degradation behavior of these materials and their hemocompatibility.

### 3.1. Preparation of the Nanofibrous Materials

The polyester materials considered in this study were made from PCL and PLCL. The selection of the polymers and the spinning conditions was aimed at allowing for the study of the influence of the chemical composition and morphology (fiber diameter) of the materials and the solvent systems used for electrospinning. A polymer with two differing molecular weights (45 and 80 kDa) and a solvent system without organic acids were used in the preparation of the PCL materials; the PLCL material (with a molecular weight of 80 kDa) was spun from two solvent systems (with and without organic acids).

**Figure 1:**
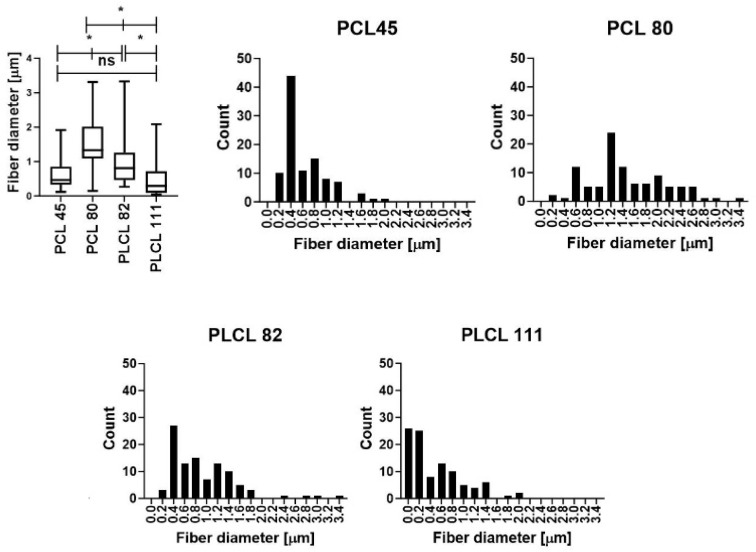
Graphs (boxplots) of the fiber diameters of the electrospun samples with histograms showing the fiber diameters. The statistical analysis was performed using the Friedman test: *n* = 100; *p* < 0.05 (ns); 0.001 (*).

**Figure 2:**
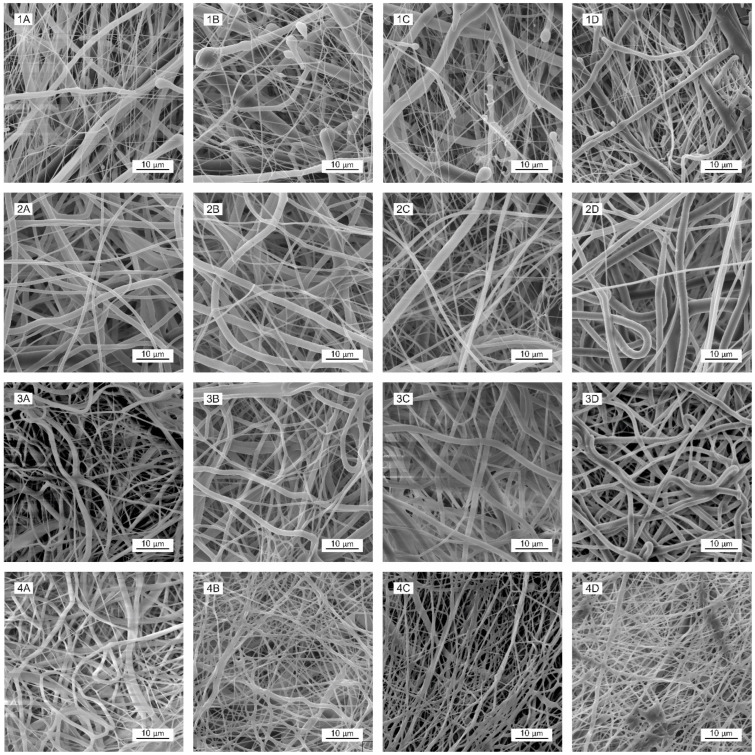
Comparison of selected SEM images of the electrospun materials without sterilization (**A**) and the same materials subjected to ethylene oxide (**B**) and gamma sterilization at doses of 15 kGy (**C**) and 40 kGy (**D**). PCL45 (1), PCL80 (2), PLCL82 (3), PLCL111 (4). Scale bar: 10 µm.

The morphology of the materials was evaluated using scanning electron microscopy (SEM) images (Figure 1). Histograms of the electrospun materials are presented in Figure 2. The results obtained revealed that the molecular weight of the polymer comprises an important parameter in terms of influencing the diameters of fiber layers prepared using electrospinning technology [47,48]. By maintaining the process conditions during the spinning process and a comparable degree of polymer solution viscosity (the optimal electrospinning viscosity of polymer solutions is an order of magnitude similar; see Table S3 App C), higher PCL molecular weights result in the production of higher-diameter fibers; see Table 2 and Figure 1. The type of solvent system comprises a further important parameter [49]. We observed a decrease in the fiber diameters of the resulting PLCL samples following the addition of acetic acid to the spinning solution. The impact of acids on PCL electrospinning was reported in a study by Ferreira et al. [50,51,52].

### 3.2. Sterilization of the Materials and the Analysis of the Materials following Sterilization

The electrospun nanofibrous materials were sterilized using gamma irradiation and ethylene oxide methods. Two different intensities, i.e., 15 and 40 kGy, were used for the gamma irradiation treatment. The impacts of the two sterilization methods on the materials were assessed in terms of changes in the material morphology (SEM and image analysis), the chemical composition (FTIR), and the molecular weight of the polymer (GPC).

### 3.3. Morphology Analysis

**Figure 3:**
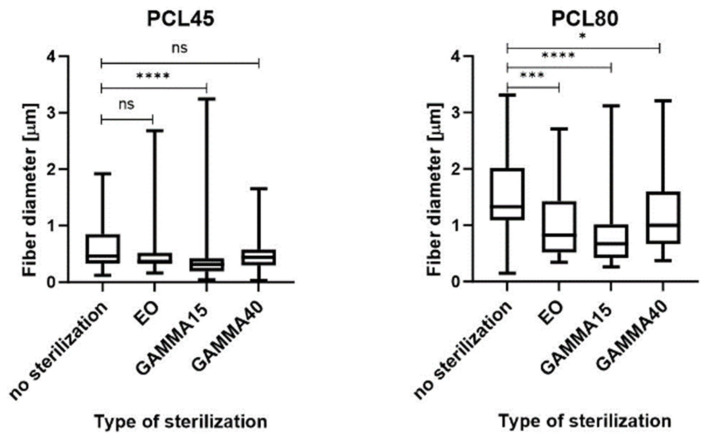

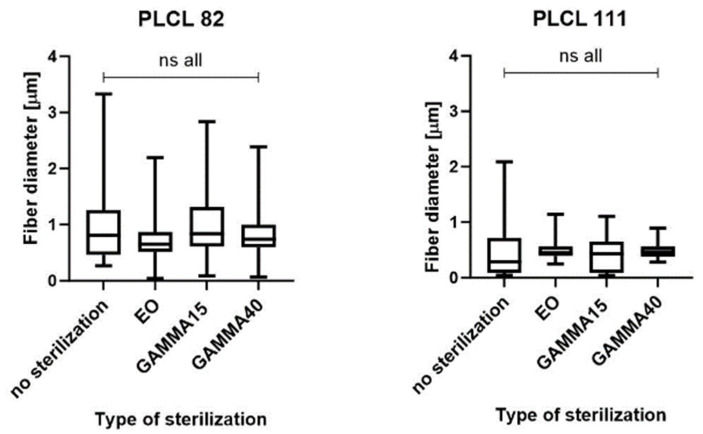
Graphs of the fiber diameters for the non-sterilized electrospun samples and the samples sterilized with ethylene oxide and gamma irradiation at 15 and 40 kGy. The statistical analysis was performed using the Friedman test: *n* = 100; *p* < 0.1234 (ns); 0.0332 (*); 0.0002 (***); 0.0001 (****).

The morphology of the materials following sterilization was evaluated via scanning electron microscopy (SEM) images (Figure 2). Fiber melting is evident in the images of the PLCL111 samples treated with ethylene oxide (Figure 2(4B)) and gamma irradiation at 15 and 40 kGy (Figure 2(4C,4D)) in contrast to the non-sterilized samples (Figure 2 SEM (4A)). Fiber melting, i.e., the apparent melting of the fibers at higher temperatures, is known to occur during the degradation process [14] and may also be caused by the action of the electron beam in the electron microscope and the sample [53]. Moreover, the PLCL111 sample evinced the lowest fiber diameters of all the samples, which may also have affected the morphology following sterilization. A further important aspect concerns the presence of acetic acid in the solvent system, which may have acted to promote fiber degradation. No significant visible changes were observed concerning the PLCL82 material following the application of either ethylene oxide or gamma irradiation (both doses) (Figure 2(3B–D)). This corresponds with the results published by Haim Zada et al. [54]. No significant visual changes were observed in the morphology for the PCL45 and PCL80 materials according to the SEM analysis following the application of either ethylene oxide (Figure 2(1B,2B)) or gamma irradiation sterilization (Figure 2(1C,D,2C,D)). The results were in agreement with the research findings of Augustine et al. and Preem et al. [55,56]. However, the subsequent fiber diameter analysis (Figure 3) revealed significantly smaller fibers for the PCL45 samples following gamma irradiation (15 kGy) and the PCL80 samples following the application of ethylene oxide and gamma irradiation than the non-sterilized samples.

No changes were determined concerning the fiber diameters of the PLCL82 and PLCL111 samples. The observed changes in the fiber diameters are likely to have been due to the inhomogeneity of the PCL materials. The images show the presence of thick and thin fibers, and the histograms reveal the dual nature of the fiber diameters. Their distributions were not homogeneous and varied in different parts of the material. Electrospinning is based on the self-organization of polymer liquids in an electric field, which may lead to a very broad fiber diameter distribution as well as inhomogeneities within the layers [57]. Very small changes in the process or material parameters, even during the electrospinning process, are known to result in significant changes in the fiber diameters.

### 3.4. Analysis of Changes in the Chemical Structure

**Figure 4:**
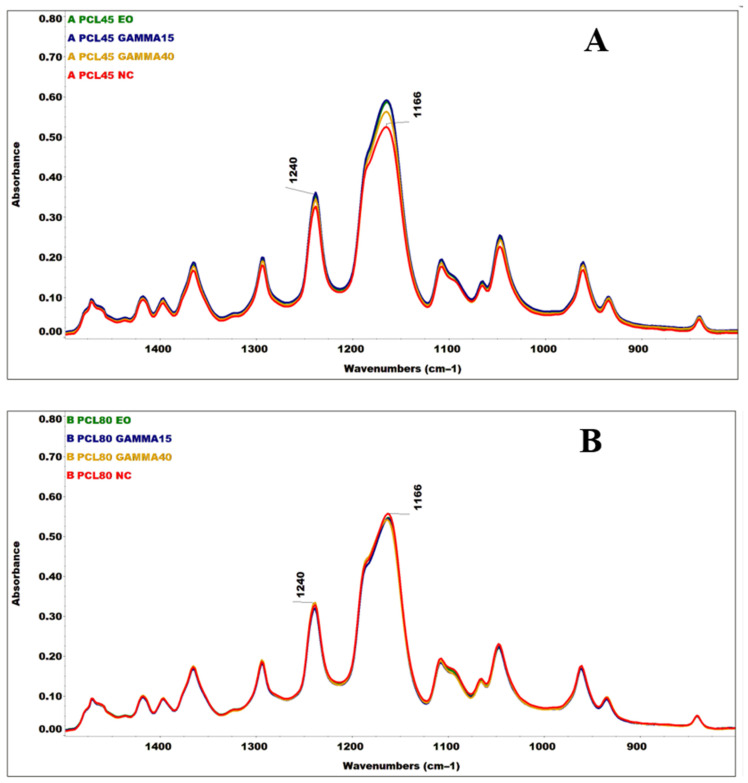

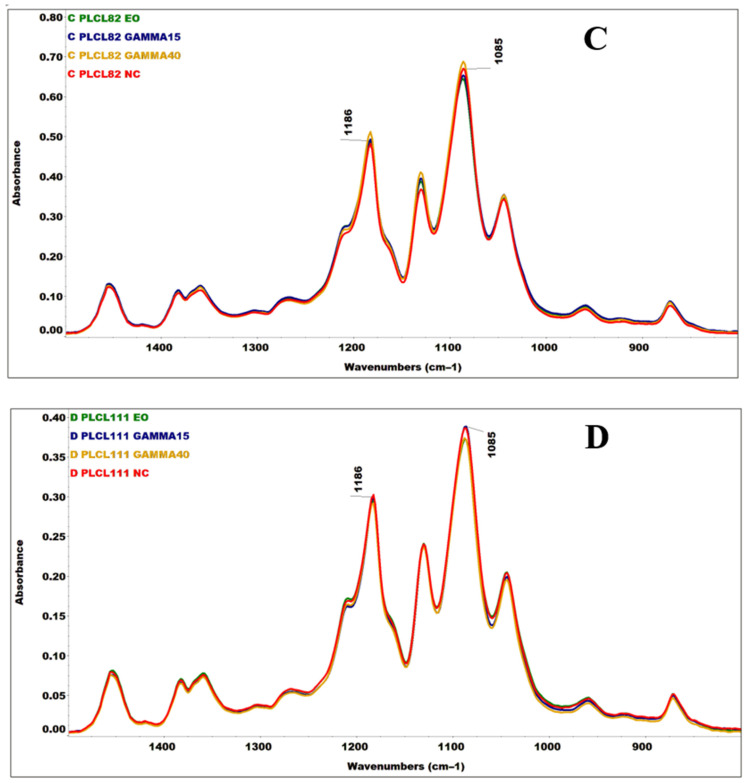
Extracts from the infrared spectroscopy (FTIR spectra) graphs of the tested nanofiber materials concern the verification of the possible formation of new bonds due to sterilization. FTIR analysis was performed for the PCL45 (**A**), PCL80 (**B**), PLCL82 (**C**), and PLCL111 (**D**) materials. Spectra are shown for the non-sterilized materials (NC) and the materials subjected to ethylene oxide irradiation and gamma irradiation at 15 kGy and 40 kGy.

An FTIR analysis was performed aimed at verifying the potential formation of new bonds due (mainly) to the sterilization method applied (Figure 4 and Figure S2).

Ionizing radiation has the potential to initiate a complex C-C, C-H, and C-O bond cleavage mechanism in polymers, which often results in the formation of radicals, the instability of which may subsequently lead to polymer crosslinking or to the shortening of the length of the polymer chain. If the polymer is exposed to ionizing radiation in the presence of oxygen [58] or to ethylene oxide, it may succumb to oxidation. PCL and PLCL polymers contain C-H bonds in their chemical structures from aliphatic chains (stretching vibrations of the C-H bonds in the 2960–2780 cm^−1^ region) that can be cleaved and oxidized to form C=O, O-C, and C-O-C bonds. These bonds are present in both PCL and PLCL [59], [60,61,62], which renders it very difficult to identify changes. All the spectra shown (Figure S2) were subsequently compared to the band intensity of the stretching vibration of the C=O carbonyl group (1722 and 1750 cm^−1^) [59,62,63]. No broadening of the C=O band was evident in the sterilized material spectra, nor were any significant changes determined in the ratio of its intensity with the C-H bands (2945, 2865, and 2870 cm^−1^) [62,63], which appears to indicate the significant oxidation of the material. However, concerning the spectra of the PCL45 and PLCL82 materials exposed to gamma irradiation, a change was evident in the relative ratios of the intensities of the C=O and C-H bands to the C-O-C bands (1240, 1166, 1186, and 1085 cm^−1^) [62,63] and the bands in their vicinities (Figure 4). Such intensity ratio changes in this region of primarily deformation, skeletal, and stretching vibrational bonds could potentially indicate structural changes in the irradiated material. Similar differences, but at much lower intensities, were determined in the spectra of the PCL80 and PLCL111 irradiated materials.

### 3.5. Analysis of Changes in the Molecular Weight

**Figure 5:**
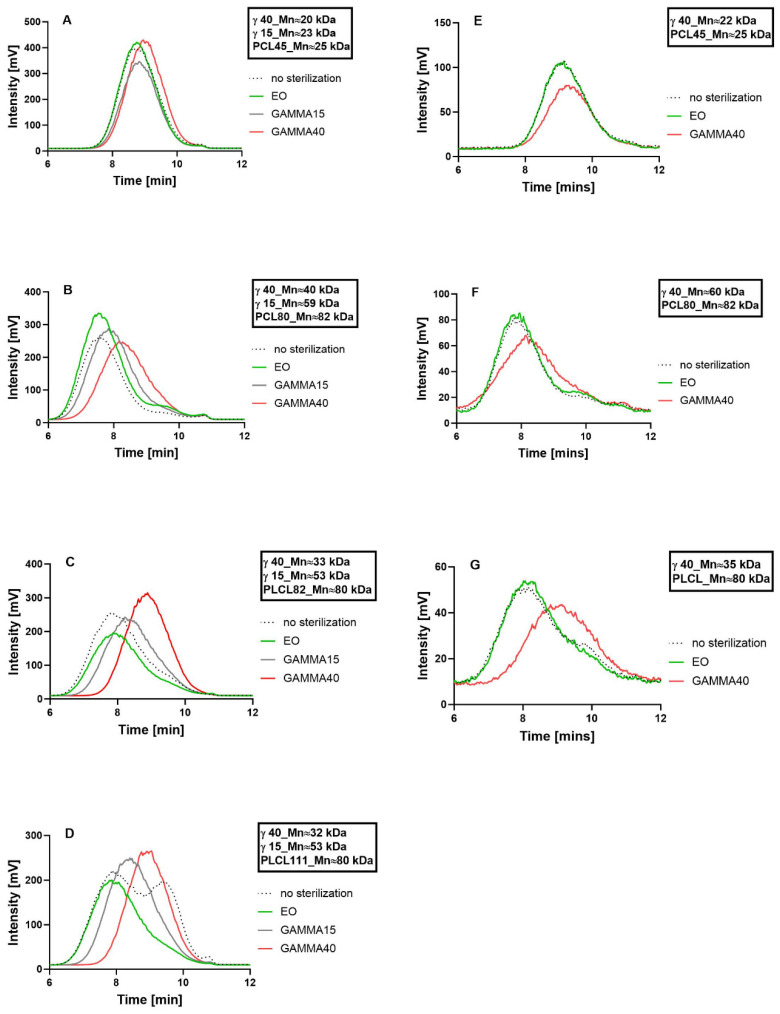
Graphs for the comparison of the GPC chromatograms of the electrospun materials and granulates from the biodegradable polyesters without sterilization and following ethylene oxide sterilization and gamma irradiation sterilization at two different doses (15 and 40 kGy): PCL45 nanofibers (**A**); PCL80 nanofibers (**B**); PLCL82 nanofibers (**C**); and PLCL111 nanofibers (**D**); PCL45 granulate (**E**); PCL80 granulate (**F**); and PLCL granulate (**G**). The data in the boxes indicate the molecular weight values calculated according to the respective standards (PS) (note: the manufacturer states M_n_ 45,000 for PCL45; however, the GPC method applied measured 25 kDa).

Changes in the molecular weights of the polymers in the electrospun nanofibrous layers before and after sterilization with ethylene oxide and gamma irradiation at intensities of 15 and 40 kGy were monitored by means of GPC (Figure 5). Decreases in the molecular weights of the polymers due to the application of gamma irradiation were observed for all the samples. The electrospun PLCL materials (Figure 5C,D) evinced more significant changes in the polymer molecular weight due to sterilization than did the PCL materials (Figure 5A,B). Gamma irradiation led to decreases in the molecular weights of both the PLCL82 and PLCL111 samples from 80 kDa to 53 kDa (to 66% of the original value), respectively, at 15 kGy and 33 kDa and 32 kDa (to 41% and 40% of the original values), respectively, at 40 kGy. Concerning PCL80, the molecular weight of the polymer decreased from 82 kDa to 59 kDa (to 72% of the original value) with the lower dose and to 40 kDa (to 49% of the original value) with the higher dose. PCL45 decreased from 25 kDa to 23 kDa (to 92% of the original value) after a dose of 15 kGy and to 20 kDa (to 80% of the original value) after the higher dose. Furthermore, concerning all the materials (except for PCL45), an increase was evident in the number of fragments with a lower molecular weight in the materials following sterilization with ethylene oxide, together with a more or less significant broadening of the molecular weight distribution of the polymer. Moreover, a peak was noticeable in the lower molecular weight fraction in the PLCL111 material without sterilization (Figure 5D).

The results clearly suggest that changes in polymer degradation are more pronounced with the increasing molecular weight of the polymer (PCL45 versus PCL80). This result corresponds to the results of a previous study on the sterilization of a nanofibrous material made from PCL with a molecular weight of 45,000, concerning which no significant change in the molecular weight of the polymer was observed following either sterilization with ethylene oxide or gamma irradiation [27], as well as to the results of studies on the sterilization of films [40,64] and fibers [65,66] made from PCL with a molecular weight of 80,000, concerning which a significant change in the molecular weight of the PCL was observed following gamma irradiation sterilization.

Polymer degradation was even more pronounced for both the PCL and PLCL polymers following gamma irradiation sterilization than following ethylene oxide treatment, which corresponds to data concerning the sterilization of PCL and PLA materials as reported by Dai et al. [24]. Moreover, an irradiation intensity dependence was evident, which corresponds to the results of studies by Bosworth et al. [65] and Di Foggia et al. [67], which evaluated the impacts of irradiation on PCL materials at intensities of 10–40 kGy and 10–50 kGy, respectively. Our results demonstrated that increasing the irradiation intensity exerts a similar effect on both PLCL and PCL. These results are consistent with those reported in a study by Chausse et al. [25]. It is also evident from the GPC results presented that certain solvent systems (an organic acid solution in the case of PLCL111, Figure 5D) lead to the degradation of polymers even without subsequent sterilization, which appears not to intensify the degradation process.

Changes in the molecular weights due to sterilization were also monitored for the polymer granulates. The graphs in Figure 5E–G clearly indicate decreases in the molecular weights following the application of gamma irradiation with an intensity of 40 kGy (the intensity of 15 kGy was not monitored), whereas no change was observed following ethylene oxide sterilization. However, the changes in the polymer molecular weights following the application of gamma 40 kGy were lower for the granulates—molecular weight (M_n_) reductions of 12% for PCL45 (from 25 kDa to 22 kDa), 27% for PCL80 (from 82 kDa to 60 kDa), and 56% for PLCL (from 80 kDa to 35 kDa)—than for the nanofibrous materials—molecular weight (Mn) reductions of 20% for PCL45 (from 25 kDa to 20 kDa), 51% for PCL80 (from 82 kDa to 40 kDa), and 59% and 60% for PLCL82 and PLCL111, respectively (from 80 kDa to 32 kDa and 33 kDa, respectively). Thus, it appears that the fiber structure influences the degrading effect of gamma irradiation, especially for PCL; moreover, this effect was most evident for PCL80 with the largest fiber diameter.

The results obtained also indicated that gamma irradiation sterilization degrades macromolecules throughout the entire volume of the material. No peak was noticeable, corresponding to the original molecular weight of the polymer. Thus, it can be reasonably stated that gamma irradiation leads to degradation within the whole volume of the material, which is characteristic of so called “bulk degradation” [14].

### 3.6. Evaluation of the Impacts of Sterilization on the Biological Behavior of the Materials—Degradation and Hemocompatibility

The sterilized materials were further tested for their degradation behavior under enzymatic-catalyzed reaction conditions. We evaluated the rate of degradation and changes in both the molecular weights of the polymers and the morphologies of the materials. The suitability of the materials for cardiovascular applications was evaluated according to the results of the hemocompatibility tests, which comprised hemolysis, thrombogenicity, and coagulation tests. Hemocompatibility was tested for all the materials within 3 months and 1 year following the sterilization of the materials.

### 3.7. Enzyme-Catalyzed Degradation of the Sterilized Materials

The experiment focused on the evaluation of the impacts of the sterilization methods applied in the study (ethylene oxide and gamma irradiation) on the degradation behavior of the materials. The degradation of the materials was assessed in terms of the degradation rate (as represented by the weight loss of the material), changes in the morphologies of the materials (the analysis of the electron microscopy images), and changes in the molecular weights of the polymers (chromatography) [11,14].

### 3.8. Analysis of the Degradation Rate (Mass Loss Analysis)

**Figure 6:**
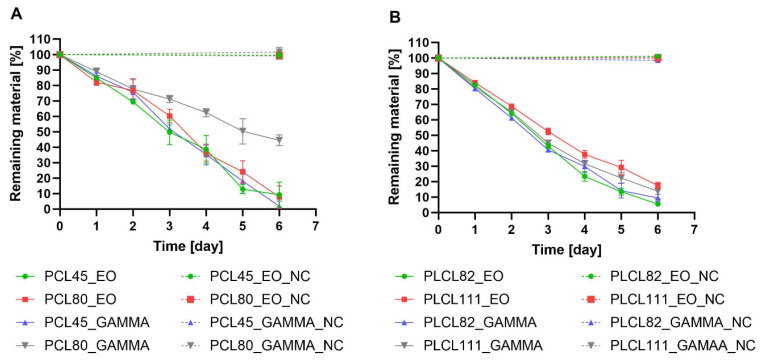
Graphs showing the results of the weight loss analysis during the enzymatically catalyzed degradation of the electrospun materials made from biodegradable polyesters: (**A**) the PCL45 and PCL80 samples after sterilization with ethylene oxide and gamma irradiation (dose of 40 kGy); (**B**) the PLCL82 and PLCL111 samples after sterilization with ethylene oxide and gamma irradiation (dose of 40 kGy). The samples marked _NC comprised sterilized materials without the addition of enzymes.

The weights of the samples of the materials were monitored over 6 days of degradation, and the weight losses were calculated using Equation (1). Due to the differing enzyme affinities of the polymer substrates, the degradation process for the PCL45 and PCL80 materials involved the use of lipase, and the degradation of the PLCL82 and PLCL111 was ensured by Proteinase K. The results of the mass loss during degradation analysis (Figure 6) indicated similar degradation trends; however, a number of differences were noted between the samples. A significant difference was observed between the PCL80 gamma and the other samples from the third day of degradation to the end of the experiment. The slowdown in the degradation process was most likely due to the higher molecular weight of the polycaprolactone in the PCL80 than in the PCL45. In addition, gamma irradiation may have influenced the creation of new chemical bonds between the polymeric chains, which may have led to the crosslinking of the polymer network in the fibers at the same time as the observed cleavage of the polymer chains [40] (Figure 6A). The weight losses of the PLCL samples as a function of time are shown in Figure 6B; the mass analysis of the samples of this material revealed very similar degradation profiles over time. However, it can be observed that, concerning the first half of the degradation experiment, the slowest degradation process was evinced by the PLCL111 ethylene oxide sample.

The mass loss analysis revealed that all the other samples degraded more rapidly (a minimal degree of variation was observed between the materials). A “break” in the degradation curves from the fourth day of degradation was common to all the samples studied, as revealed by the differing slopes of the linear trend lines in the first (days 0–3) and second (days 4–6) stages of degradation. This phenomenon can be attributed to the autocatalytic reactions of the polymer chains [68,69]. It is widely accepted that enzyme-catalyzed degradation, which includes autocatalytic reactions, acts preferentially in amorphous regions [70]. PCL is classified as a semicrystalline aliphatic polyester (with a degree of crystallinity of up to 67%) [71], whereas PLA is classified as an amorphous aliphatic polymer (with a degree of crystallinity of 20–40%) [72]. Due to the 70:30 ratio of the PLA:PCL monomers in the PLCL polymer considered in our study, this material had a lower degree of crystallinity than the PCL polymer. This factor exerted an impact on the degradation process, i.e., on the shape of the degradation curve as plotted as a function of time. A linear trend was characteristic of the semicrystalline polymers, whereas the curves that feature breaks were related to the more amorphous polymers [44,70,73].

### 3.9. Analysis of the Molecular Weight Changes following Enzyme-Catalyzed Degradation

The molecular weight changes of the sterilized (using ethylene oxide or gamma irradiation with an intensity of 40 kGy) polymer nanofibers during the enzyme-catalyzed degradation process were monitored by means of GPC. Figure 5 shows chromatograms of the nanofibrous materials during the 6 days of enzymatic degradation. In order to provide a clearer comparison, the polymer concentration was converted to the actual mass loss of the materials on a given day of degradation (e.g., concerning the third day of the degradation of EO-sterilized PLCL111, the amount of remaining material was 52.5%; the GPC values were then multiplied by 0.525).

**Figure 7:**
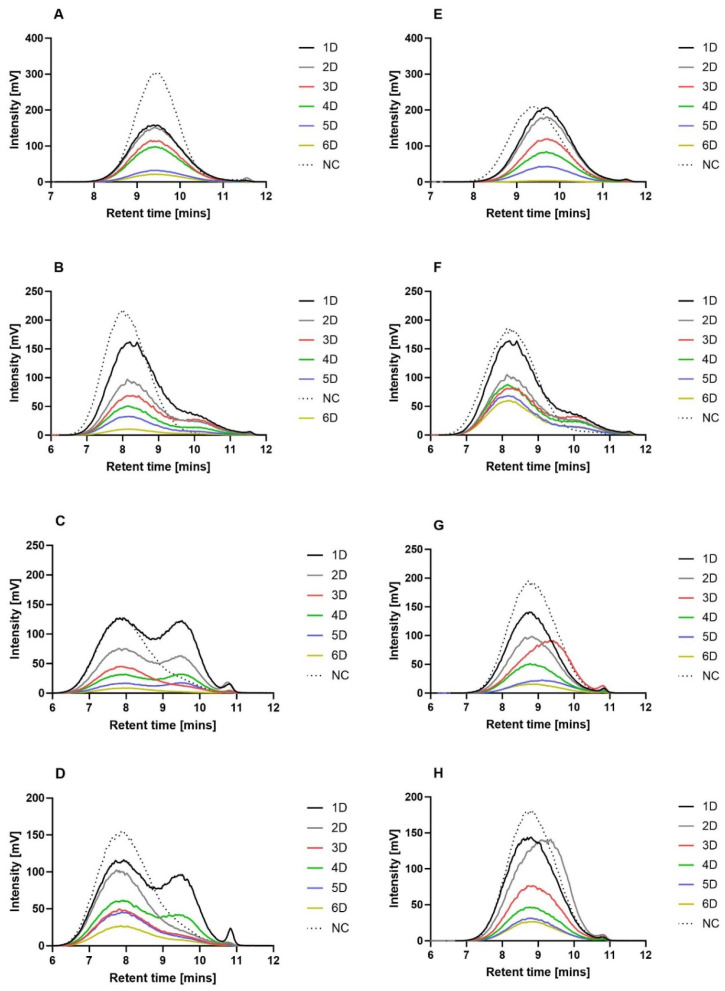
Graphs for the comparison of the GPC chromatograms of the electrospun biodegradable polyester materials subjected to ethylene oxide and gamma irradiation (dose of 40 kGy) sterilization during the degradation process. The number with the letter D indicates the number of days of degradation, and NC denotes samples without the addition of enzymes. (**A**) PCL45_EO, (**B**) PCL80_EO, (**C**) PLCL82_EO, (**D**) PLCL11_EO, (**E**) PCL45_GAMMA, (**F**) PCL80_GAMMA, (**G**) PLCL82_GAMMA, and (**H**) PLCL111_GAMMA. For the purposes of comparison, the polymer concentration was converted to the actual mass loss of the material on the given day of degradation.

A trend was not observed concerning the PCL45 treated with ethylene oxide toward a gradual decrease in the molecular weight of the polymer with the formation of undetectable low-molecular-weight degradation products (Figure 7A). No shift was apparent toward lower polymer molecular weights. Moreover, it was assumed that degradation occurs in phases (waves), as indicated by the similar intensities on days one and two and on days three and four following the significant decrease in the intensity on day three. The ethylene oxide-sterilized PCL80 material was observed to behave differently during subsequent degradation with lipase, i.e., it evinced a shift toward lower molecular weights during the degradation process in contrast to the negative control. We observed the broadening of the curves and the formation of a new peak, which indicated the presence of degradation products with lower molecular weights throughout the entire volume of the materials (bulk degradation) (Figure 7B). No wave-like degradation was observed. Distinct wave-like degradation phases were observed for the PLCL82 material treated with ethylene oxide (Figure 7C). New peaks that represented lower-molecular-weight degradation products were evident during the first and second days of degradation. These peaks disappeared on the third day of the process, followed by the complete degradation of the lower-molecular-weight products. The same trend was observed on the following days—peaks representing degradation products with lower molecular weights were observed on the fourth and fifth days of degradation but were not detected on the sixth day. Although the PLCL111 sterilized with ethylene oxide evinced a similar trend to the PLCL82 material (Figure 7D), differences were apparent in the total degradation rate of the low-molecular weight-degradation products and the smoothing of the newly formed peak due to degradation; a curve with two peaks was observed on the first day. The disappearance of the peak representing the lower molecular weight products was observed on the second and third days, accompanied by the minimal degradation of the high-molecular-weight chains. The depletion of the low-molecular-weight products, which probably degraded preferentially, was followed by the renewed degradation of the high-molecular-weight chains. A curve with two peaks was again observed on the fourth day. The final phase of the experiment consisted of the renewed preferential degradation of the low-molecular-weight products.

The gamma-irradiated PCL45 (Figure 7E) evinced a different trend than the same material treated with ethylene oxide (Figure 7A). Unlike the PCL45 material following EO sterilization, the activity of the enzymes concerning the gamma-sterilized PCL45 altered the polymer molecular weights of all the chains at the same time on the first day. The resulting shorter chains then gradually degraded to low-molecular-weight undetectable products; no shift was observed toward lower molecular weights in the graph, and the detector indicated decreases in mass loss only during the following days of degradation. The degradation behavior of the PCL80 material following gamma irradiation (Figure 7F) was observed to be similar to that of the same material following EO sterilization (Figure 7B).

The gamma-treated PCL80 evinced a decrease toward lower molecular weight chains and gradual degradation over the course of the experiment (the stretching of the curves and the formation of a smaller peak that indicated a lower molecular weight). However, a noticeable difference was observed in terms of the rate of degradation of these two materials—the gamma-sterilized PCL80 evinced a slower degradation rate, which may have been due to the cross-linking of the macromolecules in the material due to gamma irradiation in a similar way to that observed and described in a study by E. Cottam [40].

The difference in degradation of the gamma-sterilized PLCL material compared to the same material sterilized with EO was more significant than that observed for the PCL materials. No similar trend was observed between the ethylene oxide (Figure 7C) and gamma-sterilized PLCL82 materials (Figure 7G). The development of the degradation of the PLCL82 material treated with gamma irradiation was unchanged in terms of the molecular weight of the polymer, and rather than following a wave-like trend, degradation proceeded gradually without the presence of detectable low-molecular-weight products. The exception concerned the third day of the experiment, when a slight shift to a lower molecular weight was observed, which was followed by a return to the original values. The gamma-sterilized PLCL111 (Figure 7H) also behaved differently than the same material sterilized using ethylene oxide (Figure 7D) during degradation. No characteristic wave-like degradation and the formation of new peaks representing lower molecular weights were observed in the case of the gamma-treated PLCL111. In contrast, no changes were observed in the molecular weight of the polymer during degradation. The only exception concerned the second day on which a shift to a lower molecular weight was observed; however, there was practically no change in the amount of nanofibers. A return to the original molecular weight and a significant decrease in the detector response were detected on the third day, and degradation proceeded gradually with no detectable low molecular weight chains. A comparison of the timing of the shift of the peaks to lower molecular weights for the PLCL82 gamma and PLCL111 gamma materials led to the conclusion that the solvent system supplemented with acid acted to accelerate the degradation process of the PLCL nanomaterials.

It is important to note that the enzyme-catalyzed degradation data indicated differences in the degradation mechanisms of the tested materials. The degradation results for the PCL45 materials suggested the occurrence of degradation via a mechanism that acted from the surface of the fibers, with no degradation in the bulk of the material (or the proportion of degraded molecules remaining in the bulk of the material was below the detection limit of the method applied). In contrast, concerning the PCL80, fractions with a lower molecular weight were observed in the remaining bulk of the material. The results thus indicated the degradation of the PCL80 throughout the entire volume of the material (bulk degradation). The degradation mechanism (surface/bulk) is related to the rate of penetration of water into the degraded material. In cases where the penetration of water is slow, with more rapid degradation from the surface of the material, degradation within the entire volume of the material is retarded or does not occur at all [14,74,75]. The rate of water penetration is influenced by a number of properties, including the molecular weight of the polymer [75]. Moreover, since the PCL45 material had one of the smallest fiber diameters of all the tested nanofibrous materials, it is likely that the degradation behavior was also related to this parameter.

The analysis of the degradation behavior of the materials exposed to the two sterilization methods indicated changes in the degradation mechanisms of the PLCL materials according to the method used to sterilize the material. Following sterilization with gamma irradiation, the polymer retained its molecular weight during degradation in the bulk of the material, and the results of the GPC analysis corresponded to degradation from the fiber surface. However, concerning ethylene oxide sterilization, fractions of the PLCL with a lower molecular weight were observed to be present (in the form of waves) in the rest of the material, which would appear to indicate bulk degradation. This behavior was thought to be related to changes in certain properties that are important in terms of the penetration of water into the material. This factor is slowed down by the increasing degree of crystallinity of the polymer [75], as well as cross-linking [43], which has previously been mentioned as one of the possible consequences of the application of gamma irradiation [40].

### 3.10. Morphology Analysis following Enzyme-Catalyzed Degradation

**Figure 8:**
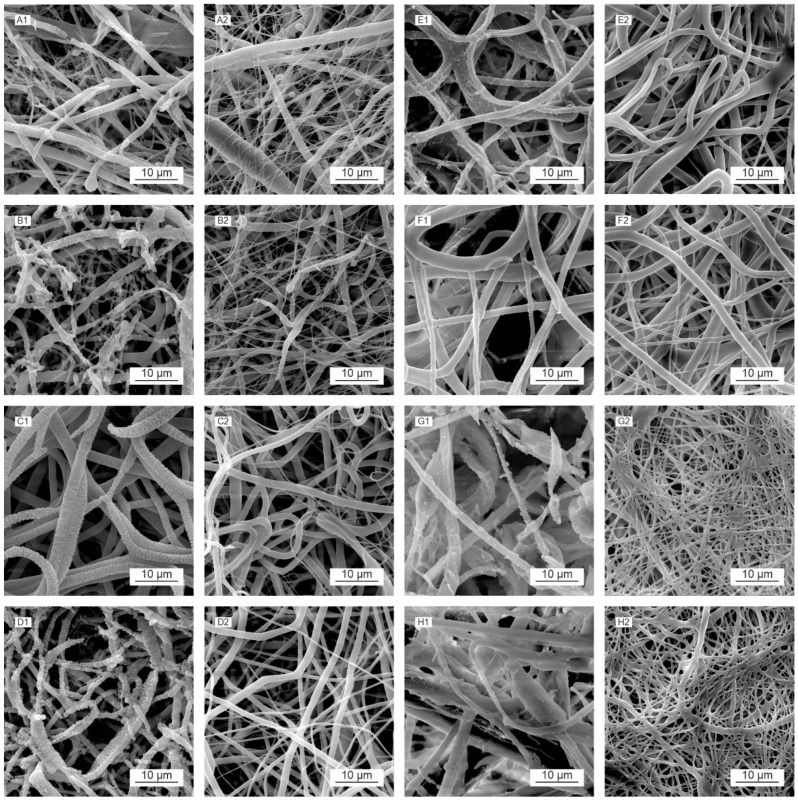
Morphological changes to the electrospun biodegradable polyester materials following sterilization (using ethylene oxide or gamma irradiation), as illustrated by the SEM images obtained during the enzymatically catalyzed degradation process on the third day of testing (1) and the negative controls (2): PCL45_EO (**A**), PCL45_GAMMA (**B**), PCL80_EO (**C**), PCL80_GAMMA (**D**), PLCL82_GAMMA (**E**), PLCL82_GAMMA (**F**), PLCL111_GAMMA (**G**), and PLCL111_GAMMA (**H**). The scale bars represent 10 µm.

The morphological changes caused by the enzyme-catalyzed cleavage of the polymer chains during the degradation process were observed by means of SEM (Figure 8). We observed morphological changes, including fiber breakage, disintegration, melting, and restructuring, during the degradation process [14]. The images in Figure 8 illustrate the various structural changes that occurred for each material and the influence of the sterilization method applied.

Compared to the negative controls (Figure 8(A2,B2)), the PCL45 samples following degradation (Figure 8(A1,B1)) exhibited the preferential loss of smaller diameter (around 400 nm) fibers at the commencement of the degradation process (first and second days), which degraded rapidly and practically disappeared over the first few days. However, the gradual restructuring of the thicker fibers was observed, accompanied in the advanced stages (from day 3) by the appearance of defects and fiber breakdown. The restructuring of the fibers was caused by the more rapid degradation of the amorphous parts of the polymer in the fiber. The free volume created by the degradation of these components allowed for the enhanced mobility of the remaining macromolecules, which rearranged themselves into crystalline (more energetically favorable) structures [14,76,77,78]. The thin parts of the fibers were observed to be completely degraded, and the fibers disintegrated completely in the advanced stage of degradation. A comparison of the PCL45 ethylene oxide (Figure 8(A1)) and PCL45 gamma (Figure 8(B1)) materials at the same time points revealed that degradation appeared to be more pronounced for the gamma-sterilized PCL45, which corresponded to the results of the GPC analysis (Figure 7A,E), via which we determined a decrease in the molecular weight of the polymer following gamma irradiation from the first day of degradation, whereas no reduction in the molecular weight was observed following EO sterilization.

PCL80 (Figure 8(C1,C2,D1,D2)) evinced higher fiber diameters than the PCL45 material (see Table 2), and degradation was observed to be more uniform from the outset, i.e., fiber restructuring appeared in most of the fibers at the same time. Needle-like formations appeared along the lengths of the fibers in addition to their characteristic, relatively regular restructuring; fiber melting was also evident for this material. A fiber-melting-like morphology is often observed in low-T_g_ amorphous fibrous materials (e.g., PLGA and PLA). Fiber melting is thought to occur when the molecular weight of the polymer has declined to a point where its T_g_ approaches the degradation temperature. The polymer chains in the amorphous phase are mobilized in such a situation, and the fibers tend to “melt” together to reduce the surface tension. Concerning materials with higher crystallinities, or T_g_ (e.g., PGA and PCL and their copolymers), the fibers tend to break during the process [14].

The advanced stage of the degradation of the PCL80 materials was characterized by the appearance of defects, massive fragmentation, and the degradation of the fibers. In terms of the applied sterilization method, degradation was more pronounced for the PCL80 gamma (Figure 8(D2)) than for the EO-sterilized PCL80 material (Figure 8(C2)). The PLCL samples (Figure 8(E1,E2,F1,F2)) were observed to behave differently than the PCL45 and PCL80 samples during degradation. Although the melting of the fibers and their restructuring were also observed during the degradation process, they were significantly less evident than for the PCL materials, especially concerning their restructuring. The loss of smaller-diameter fibers was observed for the PLCL82 (Figure 8(E1,F1)) material at the commencement of the degradation process, while surface changes were observed concerning the higher-diameter fibers. The morphological changes on the surface did not evince a geometrically defined and sharp character. The surfaces of the fibers were wrinkled, and the melting of the fibers was observed. The more pronounced restructuring of the fibers was observed only in the final stage of degradation for the gamma-sterilized PLCL82 (Figure 8(F1)). The development of the degradation of this material appeared overall to be more extensive and rapid in terms of the extent of the morphological changes than that of the ethylene oxide-sterilized PLCL82 (Figure 8(E1)). Concerning the PLCL111 samples (Figure 8G,H), we observed the pronounced melting of the fibers and the formation of bulkier polymer clusters as early as in the initial stages of degradation, in contrast to the negative controls (Figure 8(G2,H2)) and the other materials studied. This was evident for both sterilization methods. The clusters then degraded progressively, and their surfaces exhibited wrinkling and defects. The low degree of restructuring of the PLCL fibers was interesting in view of the results of a study by Horakova et al. [28], which revealed the very clear restructuring of PLCL following the ethylene oxide sterilization of the material. The difference in behavior in our study was thought to be related to the solvent system used for electrospinning, i.e., a solvent system consisting of chloroform–ethanol–acetic acid (1:1:1) was used in the study by Horakova et al. [28].

### 3.11. The Impacts of Sterilization on the Hemocompatibility of the Materials

It is essential that an assessment be performed of any material destined for use in cardiovascular applications in terms of its hemocompatibility, i.e., its interaction with selected components of human blood. Hence, this study included the determination of the influence of the studied materials on the integrity of the erythrocyte membrane (hemolysis testing), as well as on platelet activation (thrombogenicity testing) and potential anticoagulant effects (coagulation testing). The testing methodology adopted in this study was based on the procedures described in one of our previous papers [45]. Hemolysis was tested by means of direct contact with fresh, noncoagulated whole blood (blood drawn into an anticoagulation solution); thrombogenicity was tested via direct contact with fresh native platelets (PRP, platelet-rich plasma); and coagulation was assessed via direct contact with clinical plasma (thawed clinical plasma). Aiming at determining the short-term and long-term impacts of the two types of sterilization on hemocompatibility, the materials were tested shortly after sterilization (up to 3 months) and 1 year following sterilization. The testing procedure included a comparison of the two sterilization methods for each material, and evaluated the influence of the polymer used, the molecular weight of the polymer, and the solvent system used in the production of the fibers.

**Figure 9:**
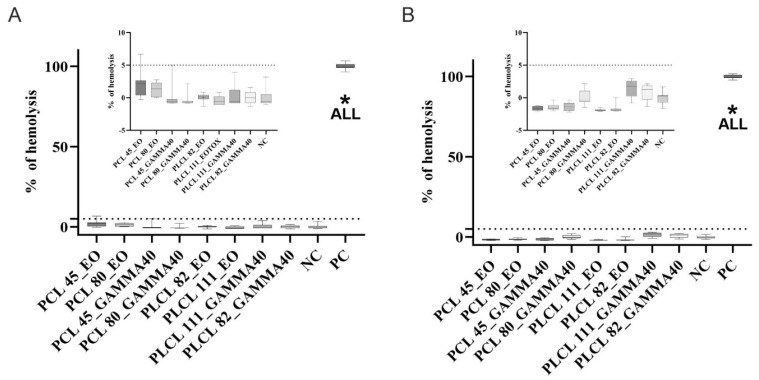
Graphs showing the percentage of hemolysis evaluated based on the measured absorbance of the hemoglobin released in the hemolysis test following the interaction of non-coagulable whole blood with the nanofibrous materials following sterilization with ethylene oxide (PCL45_EO, PCL80_EO, PLCL82_EO, and PLCL111_EO) and 40 kGy gamma irradiation (PCL45_GAMMA40, PCL80_GAMMA40, PLCL82_GAMMA40, and PLCL111_GAMMA40) 3 months (**A**) and 1 year (**B**) after sterilization. Positive control (PC): distilled water; negative control (NC); PBS buffer. A *p* value of *p* ≤ 0.05 (*) was considered statistically significant.

The results of the hemolysis tests revealed (Figure 9) that the presence of the tested materials resulted in no clinically significant damage to the erythrocyte membrane, either in the case of the materials up to 3 months following sterilization (Figure 9A) or those after one year of sterilization (Figure 9B). The degree of hemolysis was evaluated in the form of percentages as determined by a calculation based on the measured absorbance of the positive control (erythrocytes incubated in distilled water), which expressed 100% hemolysis in the given case. The absorbances measured for the samples following incubation with the materials in the buffer environment were then converted to percentages according to this value [45,79]. The highest mean values (*n* = 10) related to the ethylene oxide-sterilized PCL45 after 3 months (2%) and the gamma irradiation-sterilized PLCL samples after one year (1.4%). However, none of the values reached the clinically significant hemolysis value of 5%. This value is based on the ISO 10993-4 standard for the testing of the hemolytic effect of materials [46].

**Figure 10:**
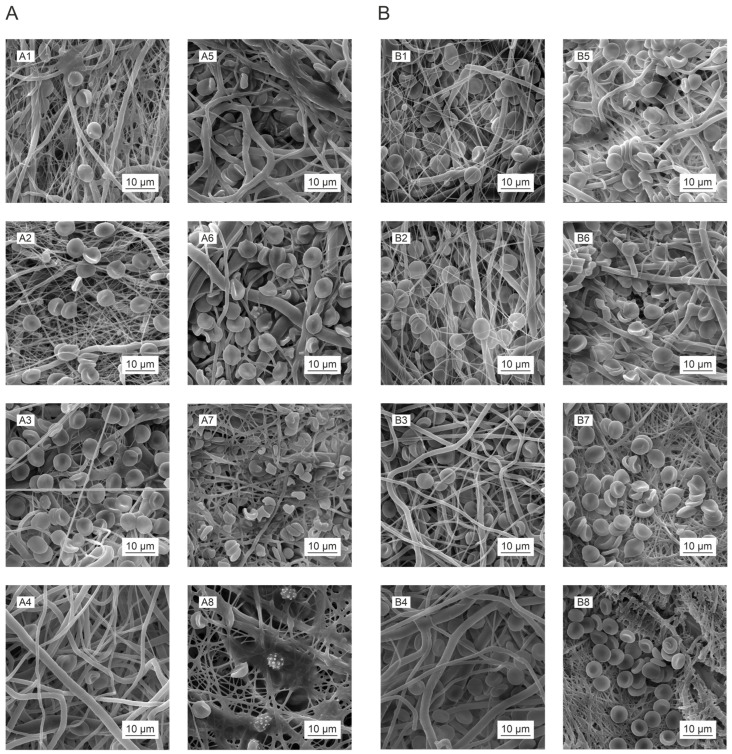
SEM images of the tested materials after incubation with whole blood (the nanofibrous materials following sterilization with ethylene oxide were tested 3 months (**A**) and 1 year (**B**) following sterilization): PCL45_EO (1), PCL45_GAMMA40 (2), PCL80_EO (3), PCL80_GAMMA40 (4), PLCL82_EO (5), PLCL82_GAMMA40 (6), PLCL111_EO (7), and PLCL111_GAMMA40 (8); scale bars: 10 µm.

However, the subsequent microscopic analysis indicated that some of the tested materials affected the behavior of the erythrocytes, changes in the morphology of which were most noticeable in the case of the PLCL111 material up to 3 months following sterilization (Figure 10(A7,A8); however, no such changes were evident in the materials tested 1 year after sterilization (Figure 10(B7,B8)). It can be assumed, therefore, that the solvent residues, e.g., the PLCL111 organic acid residues, may have affected the morphology of the erythrocytes. The noticeable degradation (especially the breakage of the fibers) of the PLCL111 and PCL80 materials one year following gamma irradiation sterilization (Figure 10(B6,B8)) is also worth noting.

Based on the results obtained, it can reasonably be stated that the tested materials do not exert a hemolytic impact, i.e., that sterilization did not lead to changes in the tested materials that led to their exerting a hemolytic effect.

**Figure 11:**
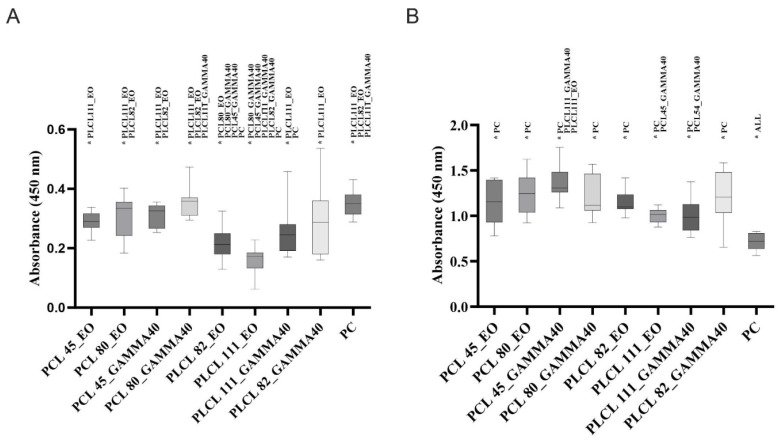
Graphs of the metabolic activity of the platelets after 2 h of incubation with the nanofibrous materials sterilized with ethylene oxide (PCL45_EO, PCL80_EO, PLCL82_EO, and PLCL111_EO) and gamma irradiation with an intensity of 40 kGy (PCL45_GAMMA40, PCL80_GAMMA40, PLCL82_GAMMA40, and PLCL111_GAMMA40) 3 months (**A**) and 1 year (**B**) after sterilization. Positive control (PC): tissue culture well-plate bottom. A *p* value of *p* ≤ 0.05 (*) was considered statistically significant.

**Figure 12:**
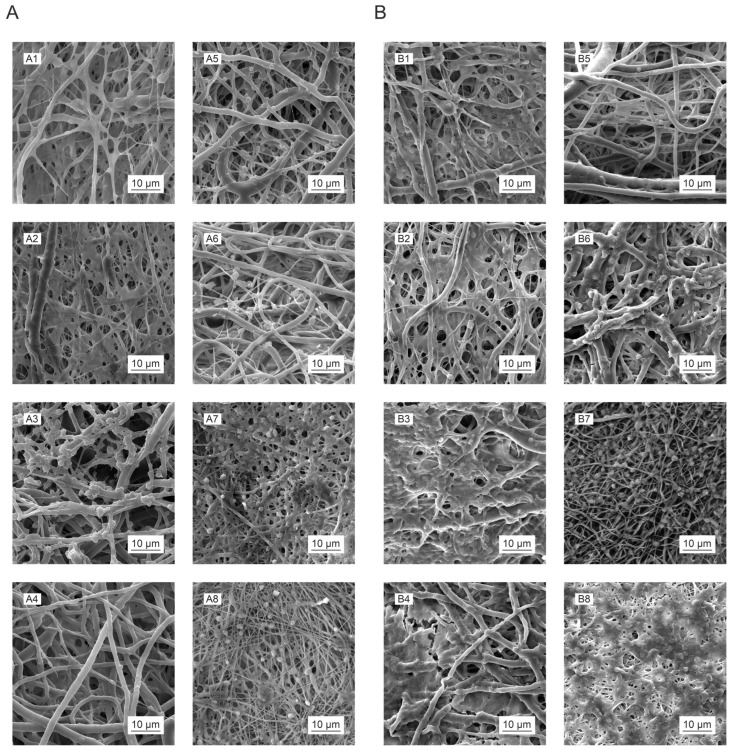
SEM images of the tested materials after 2 h of incubation with the platelets (the nanofibrous materials following sterilization with ethylene oxide were tested 3 months (**A**) and 1 year (**B**) following sterilization): PCL45_EO (1), PCL45_GAMMA40 (2), PCL80_EO (3), PCL80_GAMMA40 (4), PLCL82_EO (5), PLCL82_GAMMA40 (6), PLCL111_EO (7), and PLCL111_GAMMA40 (8); scale bars: 10 µm.

The results of the thrombogenicity tests revealed that neither of the applied sterilization methods acted to enhance the degree of platelet activation in the short term (Figure 11A); conversely, the PLCL samples following sterilization with ethylene oxide even indicated a slight suppression of platelet activation. However, the situation of the samples tested one year after sterilization differed. In this case, a significant pro-thrombogenic effect was evident with concern for all the materials (Figure 11B). The results of the platelet metabolic activity tests corresponded to those obtained from the microscopic analysis (Figure 12). Adhered and activated platelets were observed to a greater extent in the materials tested one year after sterilization than in the materials tested up to 3 months following sterilization.

This was paticulary evident for te PCL80 samples (Figure 12(B3)) and the PLCL111 samples following gamma irradiation sterilization (Figure 12(B3)). Thrombogenicity has been observed to be induced by the nanofibrous structure of materials [45], and the results of our research appear to suggest that this process is further supported by the effect of the sterilization of materials and their subsequent gradual degradation. Nanofibrous structures are interesting for the field of tissue engineering primarily due to their good interaction with cells, including platelets. The afore-mentioned study [45] considered the nanofibrous structure of these materials to be a more significant source of thrombogenicity than the chemical composition (PCL and PLCL were also included in this study). Sterilization, however, leads to changes in the molecular weight of the polymers used and related chemical changes, e.g., the frequency of the occurrence of end groups (see the FTIR analysis in the paragraph under the Section 3.4). It is possible that these changes act to further promote the activation of platelets.

The results of the measurement of the coagulation times were interesting due to the observation of the clinically significant anticoagulant impacts of some of the materials following sterilization. The prothrombin time (PT) and the activated partial thromboplastin time (aPTT) were measured as part of the test procedure. Coagulation was measured after 45 min of the incubation of the materials in clinical plasma.

**Figure 13:**
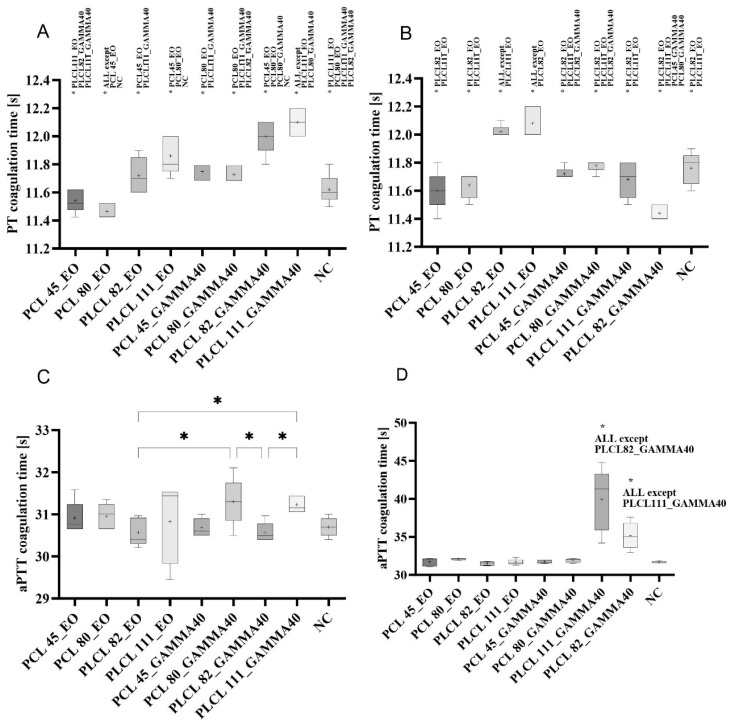
PT coagulation times (**A**,**B**) and aPTT coagulation times (**C**,**D**) measured in the coagulation tests after the interaction of clinical plasma with the nanofibrous materials following sterilization with ethylene oxide (PCL45_EO, PCL80_EO, PLCL82_EO, and PLCL111_EO) and gamma radiation at an intensity of 40 kGy (PCL45_GAMMA40, PCL80_GAMMA40, PLCL82_GAMMA40, and PLCL111_GAMMA40) 3 months (**A**,**C**) and 1 year (**B**,**D**) after sterilization. Negative control (NC): clinical plasma. A *p* value of *p* ≤ 0.05 (*) was considered statistically significant.

Although an increase in time compared to the control provides an indication of the anticoagulant effects of the material, clinically significant differences are considered for values higher than 20% of the value of the negative control [80]. In the case of the PT test (Figure 13A,B), although significant differences were observed in the measured times for some of the materials, they never attained clinically significant values. The prolonged PT times of the PLCL materials following fresh gamma irradiation sterilization are, however, worthy of note (Figure 13A), as are the slightly prolonged PT times recorded for the PLCL samples measured soon after sterilization with ethylene oxide, which were also noted 1 year after sterilization (Figure 13B). Moreover, the ethylene oxide PLCL-sterilized samples also exhibited a clear increase in these values after 1 year (Figure 13C) compared to the values measured after 3 months following sterilization.

After 3 months, the maximum measured PT times were 104% of the control value (clinical plasma) (Figure 13A, PLCL111_GAMMA40) and, after 1 year, 103% of the control value (Figure 13B, PLCL111_EO). These values do not represent significant differences from the clinical point of view, and the materials do not exert an anticoagulant effect according to the PT test [80]. The situation concerning the aPTT test differed from that of the PT tests in that a number of statistically significant differences were observed for the materials a short time following sterilization (Figure 13C); however, from the clinical point of view, they were insignificant. Conversely, the aPTT tests performed 1 year following the sterilization of the materials revealed the significant prolongation of the PLCL material times following gamma irradiation sterilization (Figure 13D). The value for PLCL82 was as high as 111% of the control value (clinical plasma), and the value was even higher for PLCL111, i.e., 126% of the control value. These values represent clinically significant differences [80], and it can be stated that the PLCL spun from a solution containing organic acids evinced anticoagulant effects one year following sterilization with gamma irradiation at an intensity of 40 kGy. A study by Horakova et al. [45] concluded that the nanofibrous structure does not exert an anticoagulant impact; therefore, we believe that this phenomenon reflected the impact of changes in the structure of the material as initiated by the gamma irradiation sterilization. Furthermore, this effect was more pronounced following the use of electrospinning solutions containing organic acids. Although, according to M. Nalezinkova [81], the used coagulation evaluation methodology has its limits (especially concerning the evaluation of the activation effect of materials), we believe that the anticoagulant behavior of PLCL materials after gamma radiation sterilization is demonstrable. Anticoagulant behavior can be caused by an increase in the adsorption of proteins present in the plasma onto the materials due to the restructuring of the surface of the fibers during their degradation. M. Nalezinkova [81] and Y. Yan [43] draw attention to this principle in their study.

## 4. Conclusions

This study included the monitoring of the impacts of sterilization on the structure and degradation behavior of selected biodegradable nanofibrous materials. Since these materials are intended for cardiovascular applications, their impacts after sterilization with concern for hemocompatibility were also monitored. The nanofibrous materials were made from two polyesters that differed, inter alia, in terms of their polymer mo-lecular weights and electrospinning conditions. Two sterilization methods were ap-plied: ethylene oxide and gamma irradiation.

The analysis of the materials following sterilization revealed the degradation of the polymers (a reduction in the molecular weight) following gamma irradiation; the degree of degradation increased in line with increases in the radiation dose. Concern-ing the PCL materials, a more significant decrease in the molecular weight was ob-served for this material as the molecular weight increased. Changes in the chemical structure of all the polymers following the application of gamma irradiation were also recorded via FTIR.

The effect of sterilization on the subsequent degradation behavior of the materials was tested using the enzymatically catalyzed degradation approach. All the materials degraded in the form of phases (or waves) following ethylene oxide sterilization, ex-cept for the PCL80 material. Whereas only a change in the rate of degradation of the polymer was observed for the PCL45, the emergence of polymers with lower molecular weights was visible in the waves for the PLCL materials. The wave-like degradation process can be explained by the fact that, in the presence of shorter molecules, degra-dation preferentially occurs up to the soluble products phase. The degradation of these short molecules is followed by the cleavage of large macromolecules. The cleaved (shorter) molecules are subsequently preferentially degraded, which acts to retard the degradation of the large molecules. Different behavior was evident in the case of the degradation of the gamma-irradiated materials. No visible wave-like degradation was observed; a clear degradation slowdown was observed for the PCL80 material, and no occurrence was evident of lower molecular weight fractions in the remaining material with concern to the PLCL materials. It can be assumed that gamma irradiation sterili-zation leads to both the cleavage of macromolecules and their cross-linking. These changes then appear to lead to a change in the properties (e.g., the crystallinity) and the related water permeability of the material. If the water permeability is retarded, the degradation of the material from the surface occurs in preference to bulk degrada-tion. Our results revealed that the degradation mechanism of the PLCL was altered as a result of the sterilization method applied. Whereas degradation occurred within the entire volume of the material following sterilization with ethylene oxide, degradation from the surface of the material predominated following gamma sterilization. Moreo-ver, the effect of the solvent system used for the electrospinning of the PLCL materials was also evident following gamma irradiation sterilization: the presence of organic acids apparently accelerates the PLCL degradation process.

The hemocompatibility tests demonstrated that the materials do not exert a he-molytic impact, even due to the changes initiated by sterilization. However, our study proved the enhanced thrombogenic properties of the materials one year following ster-ilization and indicated that the changes detected in the materials as induced by steri-lization serve to provide support for the pro-thrombogenic behavior of nanofibrous materials detected in previous studies. Our results also proved that sterilization and the changes induced by sterilization may exert a clinically significant impact on blood coagulation. The PLCL gamma-irradiated materials electrospun from organic acids were found to exert a clinically significant anticoagulant effect.

The results of our study suggest that, concerning nanofibrous materials made from degradable polyesters, it is necessary to take into account the degradation of the mate-rial and changes in the subsequent degradation behavior induced by the sterilization process. Moreover, certain changes induced by sterilization could potentially exert a clinically significant effect on the hemocompatibility of these materials. Thus, we do not recommend the use of PLCL electrospun from solutions containing organic acids and subsequently sterilized by applying gamma irradiation for cardiovascular appli-cations.

## Figures and Tables

**Table 1:** The polymer solutions prepared for electrospinning with the type of polymer, the solvent system used, and the polymer concentration, supplemented with the appropriate withdraw speed during the electrospinning of the solutions, and the areal weight of the final nanofibrous layer represented by the mean and standard deviation.

Sample	Polymer	Solvent System	Polymer Concentration (wt%)	Withdrawal Speed (mm/min)
PCL45	PCL	Chloroform–ethanol 8:2 *w*/*w*	16	40
	45 kDa			
PCL80	PCL	Chloroform–ethanol 8:2 *w*/*w*	10	20
	80 kDa			
PLCL82	PLCL	Chloroform–ethanol 8:2 *w*/*w*	10	20
PLCL111	PLCL	acetic acid–formic acid–acetone	10	20
		1:1:1 *w*/*w*		

**Table 2:** Characterization of the electrospun materials.

Sample	Polymer	Areal Weight (g/m^2^)	Fiber Diameter (µm)
PCL45	PCL 45 kDa	14.3 ± 1.8	0.62 ± 0.38
PCL80	PCL 80 kDa	17.8 ± 2.0	1.47 ± 0.67
PLCL82	PLCL	14.9 ± 1.5	0.93 ± 0.58
PLCL111	PLCL	14.4 ± 1.6	0.49 ± 0.48

## Data Availability

The data presented in this study are available on request from the corresponding authors.

## Supplementary Materials

The following supporting information can be downloaded at: https://www.mdpi.com/article/10.3390/polym16081029/s1, Figure S1: Histograms of the fiber diameter measurements for all the electrospun samples without sterilization (a) and with sterilization using ethylene oxide (b) and gamma irradiation at 15 kGy (c) and 40 kGy (d). Materials: PCL45 (1), PCL80 (2), PLCL82 (3) and PLCL111 (4); Figure S2: Infrared spectroscopy (FTIR spectra) graphs of the tested nanofiber materials compiled so as to verify the possible formation of new bonds due to sterilization. FTIR analysis was performed for PCL45 (A), PCL80 (B), PLCL82 (C) and PLCL111 (D). The spectra are presented for the non-sterilized materials (NC) and the materials subjected to ethylene oxide (EO) and gamma irradiation at doses of 15 kGy and 40 kGy (GAMMA 15 and GAMMA 40); Table S1: Viscosity of polymer solutions for electrospinning determined on a rotating viscometer.
