# Supplementary material for: The Impacts of the Sterilization Method and the Electrospinning Conditions of Nanofibrous Biodegradable Layers on Their Degradation and Hemocompatibility Behavior
Source: Polymers (Basel). 2024 Apr 9;16(8):1029. doi: 10.3390/polym16081029 (PMC11053452; doi:10.3390/polym16081029)
Supplement: Supplementary file 1 [file polymers-16-01029-s001.zip › polymers-2931534-supplementary.pdf]

S1

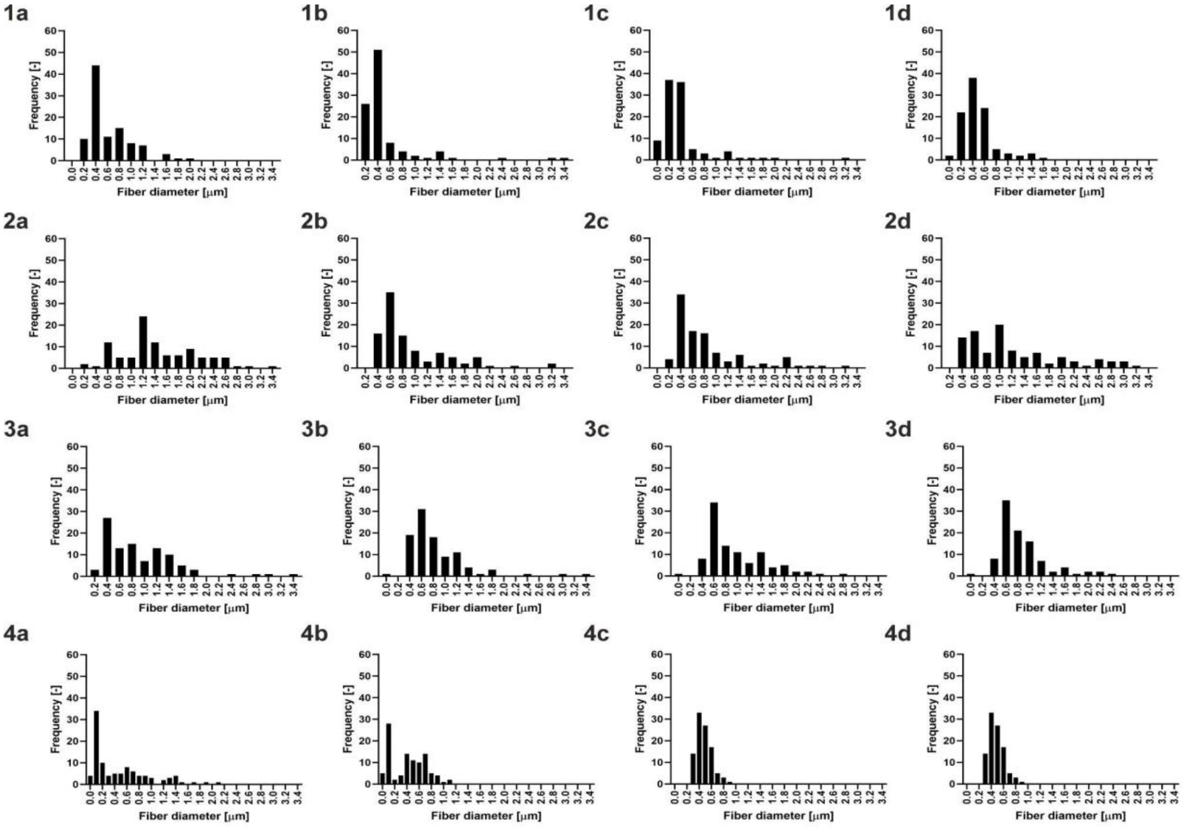

Figure S1: Histograms of the fiber diameter measurements for all the electrospun samples without sterilization (a) and with sterilization using ethylene oxide (b) and gamma irradiation at 15 kGy (c) and 40 kGy (d). Materials: PCL45 (1), PCL80 (2), PLCL82 (3) and PLCL111 (4).

S2

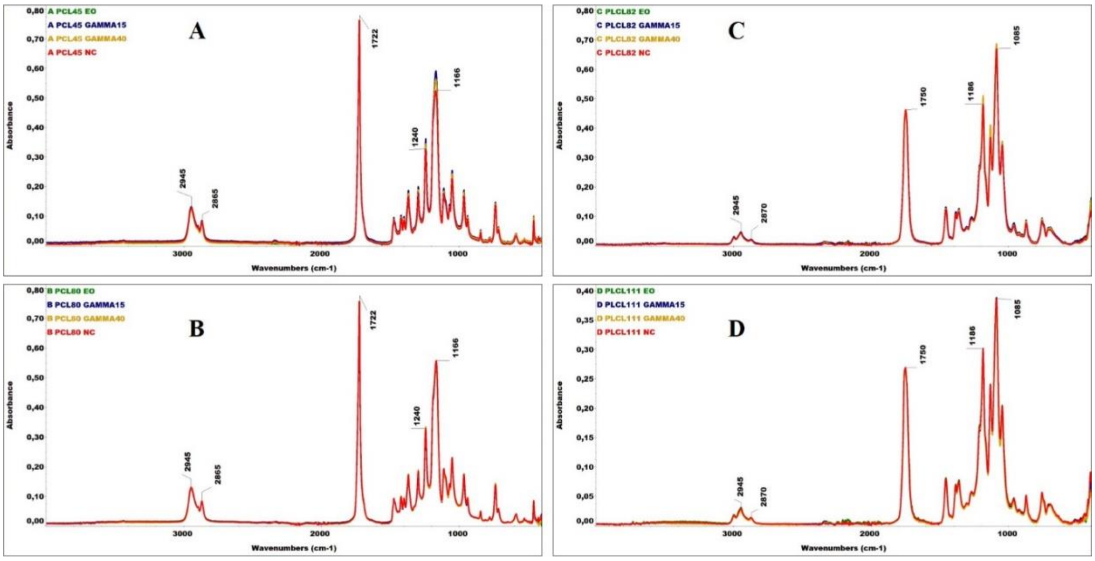

Figure S2: Infrared spectroscopy (FTIR spectra) graphs of the tested nanofiber materials compiled so as to verify the possible formation of new bonds due to sterilization. FTIR analysis was performed for PCL45 (A), PCL80 (B), PLCL82 (C) and PLCL111 (D). The spectra are presented for the non-sterilized materials (NC) and the materials subjected to ethylene oxide (EO) and gamma irradiation at doses of 15 kGy and 40 kGy (GAMMA 15 and GAMMA 40)

S3

Materials and Methods

Viscosity of Polymer Solutions

The viscosity of polymer solutions was measured on the IKA Rotavisc lo-vi Complete (IKA, Germany). The SP-2 spindle (diameter18.7 mm, immersion depth 50 mm) was used for the measurements. The volume of the solution was 50 ml. The temperature of the solution was 20.75 ± 0.5 °C. Rpm determined with respect to torque (M=50%). Triplicate measurements for each solution was made.

Table S3: Viscosity of polymer solutions for electrospinning determined on a rotating viscometer.

**Polymer Solution**	**Concentration [wt %]**	**Torque M [%]**	**rpm**	**Viscosity [mPa·s]**
PCL 45	16	50.6	82	185.67 ± 0.61
PCL 80	10	50.6	17	892.00 ± 0.85
PLCL_82	10	51.0	35	526.57 ± 0.9
PLCL_111	10	51.2	103	105. 00 ± 0.6
